# Elevated sperm DNA fragmentation is correlated with an increased chromosomal aneuploidy rate of miscarried conceptus in women of advanced age undergoing fresh embryo transfer cycle

**DOI:** 10.3389/fendo.2024.1289763

**Published:** 2024-04-08

**Authors:** Wanting Fu, Qiuying Cui, Zhiqin Bu, Hao Shi, Qingling Yang, Linli Hu

**Affiliations:** ^1^ Center for Reproductive Medicine, The First Affiliated Hospital of Zhengzhou University, Zhengzhou, China; ^2^ Henan Key Laboratory of Reproduction and Genetics, The First Affiliated Hospital of Zhengzhou University, Zhengzhou, China; ^3^ Henan Provincial Obstetrical and Gynecological Diseases (Reproductive Medicine) Clinical Research Center, The First Affiliated Hospital of Zhengzhou University, Zhengzhou, China; ^4^ Henan Engineering Laboratory of Preimplantation Genetic Diagnosis and Screening, The First Affiliated Hospital of Zhengzhou University, Zhengzhou, China

**Keywords:** sperm DNA fragmentation, chromosomal abnormality, miscarriage, chromosomal microarray analysis, assisted reproductive technology

## Abstract

**Background:**

Male sperm DNA fragmentation (SDF) may be associated with assisted reproductive technology (ART) outcomes, but the impact of SDF on the occurrence of aneuploid-related miscarriage remains controversial.

**Methods:**

Genome-wide single-nucleotide polymorphism-based chromosomal microarray analysis was performed on 495 miscarried chorionic villus samples undergone IVF/ICSI treatment from the Reproductive Medicine Center of the First Affiliated Hospital of Zhengzhou University. SDF was assessed using sperm chromatin structure assay. Patients were divided into four groups according to embryo transfer cycle type and maternal age, and the correlation between SDF and chromosome aberration was analyzed. A receiver operating characteristic (ROC) curve was utilized to find the optimal threshold.

**Results:**

Total chromosomal aneuploidy rate was 54.95%, and trisomy was the most common abnormality (71.32%). The chromosomally abnormal group had higher SDF than the normal group (11.42% [6.82%, 16.54%] vs. 12.95% [9.61%, 20.58%], *P* = 0.032). After grouping, elevated SDF was significantly correlated with an increasing chromosome aneuploidy rate only in women of advanced age who underwent fresh embryo transfer (adjusted odds ratio:1.14 [1.00–1.29], adjusted-*P* = 0.045). The receiver operating characteristic curve showed that SDF can predict the occurrence of chromosomal abnormality of miscarried conceptus in this group ((area under the curve = 0.76 [0.60–0.91], *P* = 0.005), and 8.5% was the optimum threshold. When SDF was ≥ 8.5%, the risk of such patients increased by 5.76 times (adjusted odds ratio: 6.76 [1.20–37.99], adjusted-*P* = 0.030).

**Conclusion:**

For women of advanced maternal age undergoing fresh embryo transfer, older oocytes fertilized using sperm with high SDF in IVF/ICSI treatment might increase the risk of chromosomal abnormality in miscarried conceptus.

## Introduction

The incidence of spontaneous abortion after assisted reproductive technology (ART) pregnancy is about 15%–20%, with approximately 80% occurring within the first 12 weeks (1). Embryonic chromosomal abnormalities, observed in nearly 50%–60% of miscarried tissues, are the most common cause of early miscarriage (2). Advanced maternal age and metabolic disorders are independent risk factors of chromosomal aneuploidy (CA) in embryos (3, 4), however, recent studies discovered male factors may also be associated with embryonic aneuploidy (5).

Male factors account for 40%–50% of infertility cases (6), some of which are well-defined, such as sperm malformation, low semen volume, and low sperm motility (7, 8). However, a substantial proportion of the causes remain unknown, and genetic factors are thought to be the most likely candidates (9). Genomic imprinting studies of miscarried chorionic villus samples (CVS) have shown that various paternally expressed genes play significant roles in fetal growth (10), and the integrity of sperm, especially genome integrity, is of great importance for fertilization, embryo formation and development.

Sperm DNA is more concentrated than that of somatic cells to better resist physical and chemical denaturation; however, heat, radiation, oxidative stress and abnormal apoptosis may damage DNA and lead to sperm fragmentation (11). Human spermatozoa do not have DNA repair activity (DRA) which depends on transcripts stored during oocyte maturation in the process of fertilization (12). However, the repair capacity of oocytes largely depends on their quality and the degree of sperm DNA fragmentation, and the fraction above the repair threshold may be transferred to the embryo and have genetic significance for chromosomal aneuploidy, especially in advanced women (≥ 35 years old) (13–15). Accumulating evidence indicates that high sperm DNA damage directly affects male infertility, which is closely related to poor embryo quality, low pregnancy rate and high miscarriage rate (16–19). A high sperm DNA fragmentation (SDF) index seems to reflect high chromosome abnormalities to a certain extent, thus increasing the risk of aneuploid embryos (5, 20, 21). Although the SDF test has shown potential in predicting male fertility, dissenting voices remain (22). To date, evidence of relationship between SDF level, embryonic euploidy and morphological grading is lacking. Studies on the association between SDF and embryonic chromosomal status are limited. Previous studies have mostly concentrated on the effect of SDF on clinical and laboratory outcomes after ART, but few studies have focused on the association of SDF with aneuploidy in aborted conceptus. Whether male SDF is associated with aneuploidy in miscarriage products and whether SDF can be considered as an evaluation of male infertility and aneuploidy-related miscarriage remains a focus of clinicians. Furthermore, unlike previous studies on fresh cycles, a recent study has indicated that SDF is only associated with fertilization rate but not with laboratory and clinical outcomes in frozen embryo transfer (FET) cycles (23). With the development and prevalence of freeze-all strategy, some advantages of FET cannot be ignored, so comprehensive understanding whether SDF exerts a different impact on embryo aneuploidy in fresh and frozen cycles is also an interesting topic of clinicians’ concern.

Chromosomal microarray analysis (CMA) based on genome-wide single-nucleotide polymorphism (SNP) arrays provides higher resolution and detection of copy number variation, and a higher detection rate of abnormal chromosomes in CVS (24, 25). The aim of our study was to investigate the role of paternal SDF, evaluated using the sperm chromatin structure assay (SCSA), in chromosomal aberration-related early spontaneous miscarriage after SNP array analysis for CVS in early miscarried patients who underwent *in vitro* fertilization (IVF)/intracytoplasmic sperm injection (ICSI) treatment in our center.

## Materials and methods

### Study cohort and inclusion criteria

**Figure 1:**
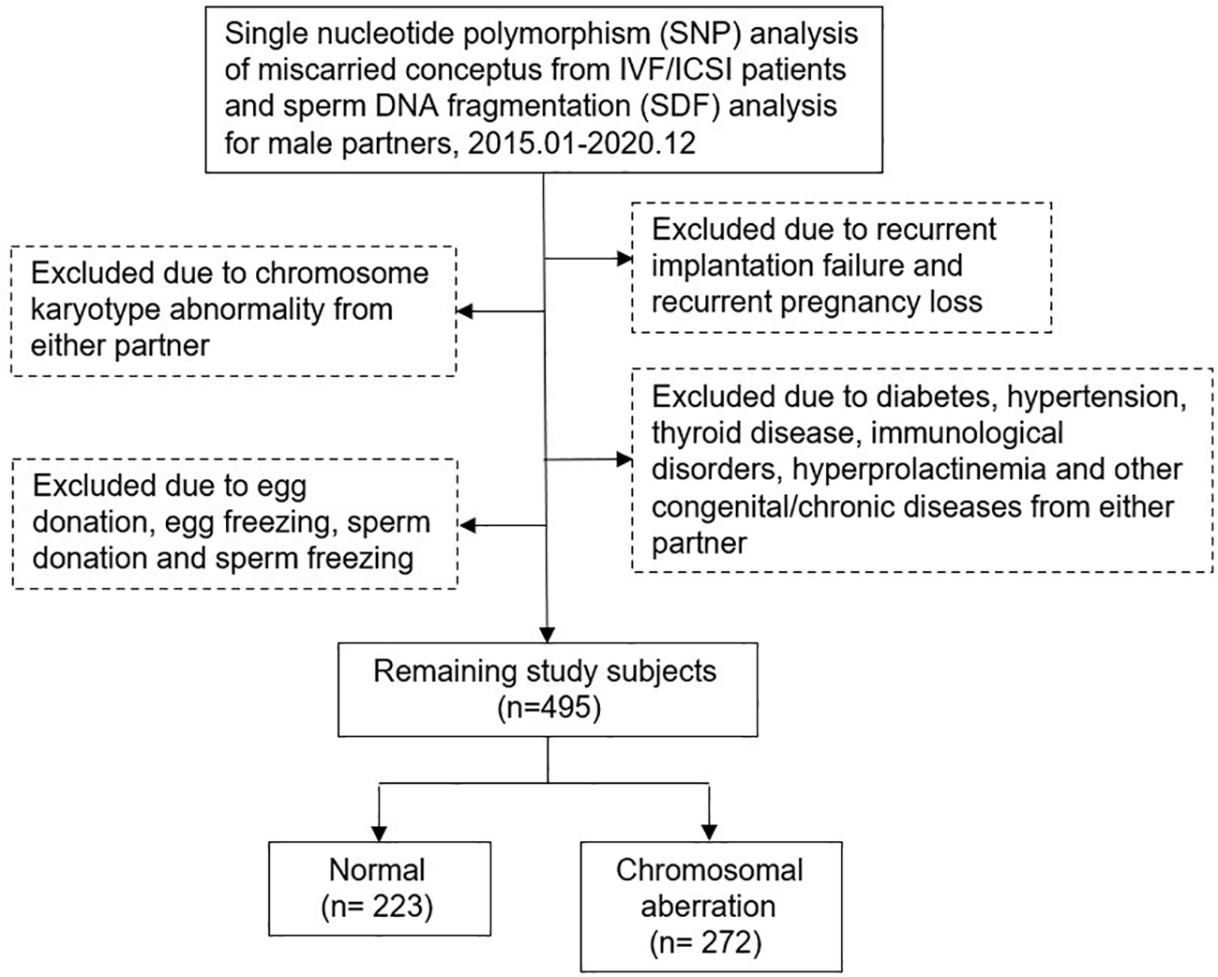
Flow chart of study inclusion and exclusion.

This study retrospectively included couples treated at our Reproductive Medicine Center from January 2015 to December 2020. All men underwent SDF testing and all women had spontaneous abortion within 12 weeks of gestation after IVF/ICSI treatment and received dilation and curettage therapy. Miscarried CVS were genetically analyzed at the Center for Preimplantation Genetic Diagnosis. Patients with chromosomal abnormalities, recurrent pregnancy loss, recurrent implantation failure, sperm freezing or donation, egg freezing or donation, immunological disorders, endocrine diseases, infectious disease, congenital or chronic diseases, varicocele and medication in men after SDF testing were excluded. Data were obtained from the Clinical Reproductive Medicine Management System/Electronic Medical Record Cohort Database (CCRM/EMRCD) in the Reproductive Medicine Center of the First Affiliated Hospital of Zhengzhou University and Henan Key Laboratory of Reproduction and Genetics. The specific inclusion and exclusion criteria were shown in **Figure 1**; 495 couples were included in this cohort analysis. All clinical and laboratory information of the couples were reviewed to evaluate the relevance between SDF level and CVS chromosomal abnormality in patients with early miscarriage. This study was approved by the Ethics Committee of the First Affiliated Hospital of Zhengzhou University (No.2023-KY-0519-002), and written informed consent was obtained from all patients at the first consultation.

### ART protocols

Both IVF and ICSI are applied for ovarian stimulation with gonadotropin-releasing hormone (GnRH) agonists according to the body mass index (BMI) and ovarian function of the patients to stimulate follicle growth and prevent premature LH surges and gonadotropins. Human chorionic gonadotropin (hCG) was administered depending on serum FSH, LH, E2 and P levels when the diameter of the largest follicle was > 20 mm and more than 2/3 of the follicles were > 16 mm. Egg retrieval was performed after 36–38 h under ultrasound guidance. The protocols of the different embryo transfer (ET) strategies were based on those stated in our published article (26) and serum β-hCG level was monitored on days 14 and 18. Clinical pregnancy was defined as the presence of intrauterine gestational blastocyst buds and primitive cardiac tube beats at 35 days after transplantation. Early spontaneous miscarriage was defined as the absence of the intrauterine fetal heartbeat on ultrasound within 12 weeks of confirmed clinical pregnancy.

### SNP array analysis

Genetic analysis of CVS in all early spontaneous miscarried patients were performed at the Preimplantation Genetic Diagnosis Center. CVS samples were cleaned and thoroughly separated from villus tissues and decidua by technicians follow the standard procedures to avoid contamination of genetic tissue (27). Samples were stored at -80°C and total villus DNA was extracted with All Prep DNA Mini Kit (Qiagen, Germany). SNP arrayed using Human CytoSNP-12v.21 Array (Illumina, San Diego, California, USA) and the data were analyzed using Genome-Studio (Illumina 2011) and Karyo-Studio V1.4 (Illumina 2011). Candidate pathogenic CNVs were mapped and identified based on the Database of Genomic Variation (DGV) (http://dgv.tcag.ca/dgv/app/faq). At least two independent technicians analyzed the data with rigorous criteria.

### SDF

Semen, obtain by masturbation after 3–7 days of abstinence from all male partners, was collected in dry sterile containers and placed in a 37°C thermostat. All examinations were completed within 1 h of sperm collection. Semen analysis was performed according to the World Health Organization standard protocol (28); sperm concentration and viability were assessed according to the instructions of the Makler counting chamber (Sefi Medical Instruments, Israel).

SCSA was utilized to evaluate SDF. All assays were performed strictly according to the instruction of the fragmentation assay kit (Cellpro Biotech, Zhejiang, China), and fluorescence was measured with a BD FACS Canto II flow cytometer (Becton Dickinson, USA). Upon excitation in the flow cytometer, intact DNA emitted green fluorescence, whereas denatured DNA emitted red fluorescence. Then SDF was expressed as the ratio of the intensity of red to the total fluorescence.

### Statistical analysis

SPSS 26.0 (IBM Corp., Armonk, NY, USA) was utilized for statistical analyses. Normality of distribution was assessed using Q-Q plots and Kolmogorov-Smirnov test. Mean ± standard deviation and 95% confidence intervals (CI) were utilized to represent continuous variables, and Student’s t-test or one-way ANOVA was applied for comparison between groups. Categorical variables were expressed as frequencies and percentages and Chi-square test, Fisher’s exact test and Bonferroni-adjusted test were applied for comparison. Univariate and multivariable Logistic regression analysis was constructed to identify risk factors for early abortion associated with chromosomal abnormalities. Odds ratio (OR) and adjusted odds ratio (a-OR) with 95% confidence intervals (CI) were calculated. The receiver operating characteristic (ROC) curve was constructed to observe predictive values of risk factors and determine the optimal threshold. All tests were two-side and statistical significance was set at P < 0.05.

## Results

### Study cohort characteristics

Demographic characteristics of the study couples, sperm parameters and baseline clinical laboratory results were presented in **Table 1**. Maternal age (33.33 ± 5.34 vs. 30.73 ± 3.83, *P* < 0.001) and paternal age (33.86 ± 6.44 vs. 31.20 ± 4.24, *P* < 0.001) in the chromosome aberration group were significantly higher than that in the normal group. Except for the number of antral follicle (14.21 ± 6.60 vs. 16.04 ± 5.82, *P* = 0.001) and AMH (3.93 ± 3.14 vs. 4.53 ± 3.42, *P* = 0.043), none of the maternal-related parameters such as BMI, infertility year, E2 and progesterone levels on trigger day showed a marked difference between two groups. For male semen examination, chromosomal aberration-related abortion group showed a higher SDF index (11.42% [6.82%, 16.54%] vs. 12.95% [9.61%, 20.58%], *P* = 0.032), whereas ejaculatory abstinence, progressive sperm motility, seminal volume and concentration were similar between two groups. Furthermore, compared with the normal group, the abnormal group had higher secondary infertility rate (60.00% vs. 50.45%, *P* = 0.034), fresh cycle rate (55.88% vs. 39.01%, *P* < 0.001), cleavage-stage D3 ET rate (58.09% vs. 41.26%, P < 0.001), and double-embryo transfer rate (52.94% vs. 43.50%, *P* = 0.037).

**Table 1:** Basic clinical characteristics of the patients.

Variable	Normal	Chromosomal Aberration	*P*
Miscarriage cycles, n	223	272	
SDF, %	11.42 (6.82, 16.54)	12.95 (9.61, 20.58)	0.032*
Maternal age, y	30.73 ± 3.83	33.33 ± 5.34	<0.001*
Maternal BMI, kg/m2	23.79 ± 3.25	23.38 ± 3.42	0.171
Infertility year, y	3.36 ± 2.41	3.80 ± 3.06	0.306
Infertility type			
Primary infertility	109 (49.55%)	108 (40.00%)	
Secondary infertility	111 (50.45%)	162 (60.00%)	0.034*
Infertility factor			
Tubal factor	110 (49.33%)	133 (48.90%)	
PCOS	28 (12.56%)	19 (6.99%)	0.075
Other	71 (31.84%)	97 (35.66%)	0.546
Male factor	14 (6.28%)	23 (8.46%)	0.398
E2 on trigger day, pmol/L	1242.25 ± 1835.37	1076.30 ± 1556.93	0.919
Progesterone on trigger day, pg/ml	0.72 ± 0.96	0.65 ± 0.83	0.095
AMH, pmol/L	4.53 ± 3.42	3.93 ± 3.14	0.043*
Number of AFC	16.04 ± 5.82	14.21 ± 6.60	0.001*
Paternal age, y	31.20 ± 4.24	33.86 ± 6.44	<0.001*
Ejaculatory abstinence, d	4.34 ± 1.16	4.34 ± 1.21	0.964
Seminal volume, mL	2.74 ± 1.28	2.53 ± 1.21	0.061
Seminal concentration, 10^6^/mL	45.41 ± 26.68	47.91 ± 34.00	0.372
Progressive sperm motility, %	38.29 ± 13.54	36.55 ± 14.50	0.173
MII rate, %	84.66 ± 13.17	85.22 ± 14.59	0.767
Fertilization method			
IVF	173 (77.58%)	219 (81.11%)	
ICSI	50 (22.42%)	51 (18.89%)	0.334
Fertilization rate, %	80.00 ± 17.85	79.25 ± 17.20	0.748
2PN rate, %	82.19 ± 17.62	84.99 ± 16.89	0.228
High-quality embryos at D3 rate, %	74.75 ± 20.39	76.35 ± 22.70	0.587
Blastocyst rate, %	54.31 ± 34.58	52.84 ± 34.16	0.776
High-quality blastocyst rate, %	39.63 ± 29.76	33.43 ± 34.84	0.331
Cycle type			
Fresh ET cycle	87 (39.01%)	152 (55.88%)	
Frozen ET cycle	136 (60.99%)	120 (44.12%)	<0.001*
ET days			
D3	92 (41.26%)	158 (58.09%)	
D5/D6	131 (58.74%)	114 (41.91%)	<0.001*
Number of transferred embryo			
1	126 (56.50%)	128 (47.06%)	
2	97 (43.50%)	144 (52.94%)	0.037*

The data were presented as mean ± standard deviation, median (P_25_, P_75_) and percentage. The difference was analyzed by Student’s t-test, Mann-Whitney U-test or chi-square test, and only P < 0.05 was considered statistically significant. Bold fonts and * Statistically significant. SDF, sperm DNA fragmentation; BMI, body mass index; PCOS, polycystic ovary syndrome; AMH, anti-mullerian hormone; AFC, antral follicle; IVF, in vitro fertilization; ICSI, intracytoplasmic sperm injection; ET, embryo transfer.

### SNP array analysis results on miscarried chorionic villi

**Figure 2:**
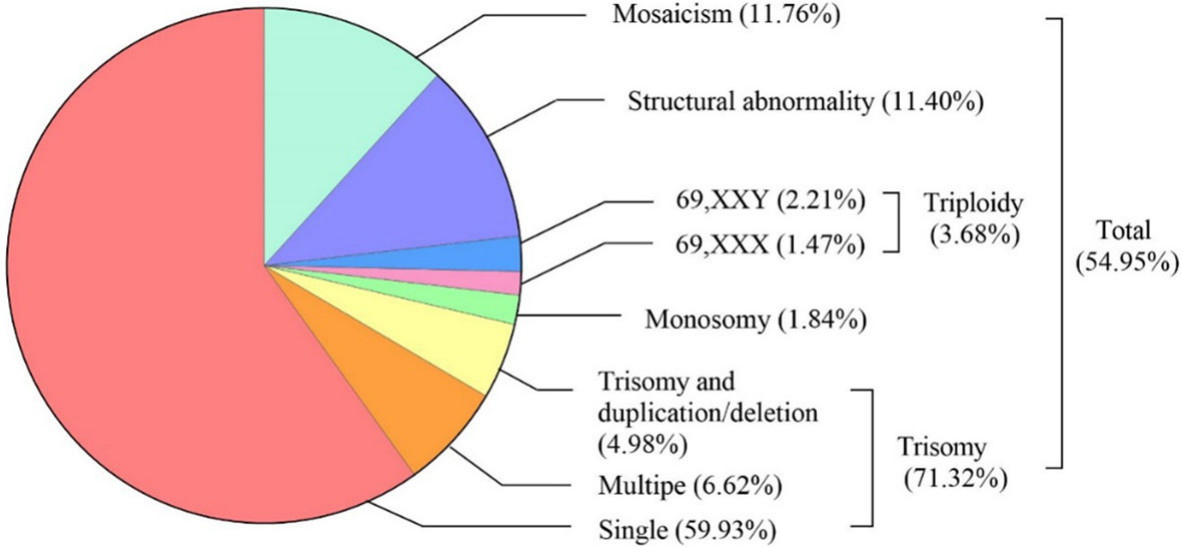
Spectrum of abnormal chromosomal karyotype in miscarried conceptus: type and Frequency.

The types and frequencies of abnormal chromosomal karyotypes in miscarried conceptus were shown in **Figure 2**. SNP array analysis revealed chromosomal aberrations in 54.95% (272/495) of miscarried CVS. The most frequent was trisomy, accounting for 71.32% (194/272) of the total aberrations, of which 84.02% (163/194) were single-chromosome trisomy, 9.28% (18/194) were polysomic trisomy, and 6.70% (13/194) were trisomy accompanied by microduplications and microdeletions. Monosomy and triploidy accounted for 2.94% (8/272) and 4.78% (13/272) of all abnormalities. The proportions of chromosome karyotype 69,XXX and 69,XXY in triploid aberration was 40% (4/10) and 60% (6/10), respectively, and chromosomal structural abnormalities and mosaicism accounted for 11.40% (31/272) and 11.76% (32/272), respectively.

### Correlations between variables and CA risk in miscarried conceptus

Univariate and multivariate logistic regression analyses were applied to evaluate the effect of variables on the CA rate (**Table 2**). After adjusting maternal age, paternal age, infertility type, ET days, fertilization method, number of transferred embryos, embryo quality, cycle type, AMH, number of antral follicle, ejaculatory seminal volume, seminal concentration and progressive sperm motility, there was a significant relationship between SDF and CA rate of miscarried conceptus (a-OR: 1.03 [1.00–1.05], a-*P* = 0.036). Advanced maternal age (≥ 35 years old) was obviously associated with the elevated CA rate (a-OR: 2.97 [1.68–5.25], a-*P* < 0.001). When 30–39 years was regarded as the reference of paternal age, male age of ≥ 40 years also significantly related to increased CA rate in the adjusted model (a-OR: 2.83 [1.18–6.79], a-*P* = 0.020). Moreover, patients undergoing frozen cycle showed a lower odds of chromosome aberrations-related miscarriage (a-OR: 0.57 [0.35–0.86], a-*P* = 0.008).

**Table 2:** Multivariable Logistic analyses of factors related to chromosomal aneuploidy in miscarried conceptus.

Variable	Crude model		Adjusted model	
	OR (95% CI)	*P*	a-OR (95% CI)	a-*P*
SDF	1.02 (1.00, 1.04)	0.018*	1.03 (1.00, 1.05)	0.036*
Maternal age				
< 35 y (n=354)	Reference		Reference	
≥ 35 y (n=141)	4.21 (2.68, 6.59)	<0.001*	2.97 (1.68, 5.25)	<0.001*
Paternal age				
20-29 y (n=151)	0.96 (0.65, 1.42)	0.831	1.30 (0.84, 2.01)	0.249
30-39 y (n=284)	Reference		Reference	
≥40 y (n=60)	4.73 (2.31, 9.89)	<0.001*	2.83 (1.18, 6.79)	0.020*
Infertility type				
Primary infertility (n=217)	Reference		Reference	
Secondary infertility (n=273)	1.47 (1.03, 2.11)	0.034*	1.06 (0.70, 1.60)	0.788
ET days				
D3 (n=250)	Reference		Reference	
D5/D6 (n=245)	0.51 (0.35, 0.73)	<0.001*	0.62 (0.34, 1.13)	0.117
Fertilization method				
IVF (n=392)	Reference		Reference	
ICSI (n=101)	0.81 (0.52, 1.25)	0.334	0.83 (0.49, 1.41)	0.494
Number of transferred embryo				
1 (n=254)	Reference		Reference	
2 (n=241)	1.46 (1.02, 2.09)	0.037*	0.90 (0.53, 1.55)	0.709
Embryo quality				
High quality (n=411)	Reference		Reference	
Non-high quality (n=84)	0.74 (0.46, 1.19)	0.215	1.27 (0.71, 2.26)	0.417
Cycle type				
Fresh cycle (n=239)	Reference		Reference	
Frozen cycle (n=256)	0.51 (0.35, 0.72)	<0.001*	0.57 (0.35, 0.86)	0.008*
AMH	0.95 (0.89, 0.99)	0.047*	1.03 (0.95, 1.11)	0.430
Number of AFC	0.95 (0.93, 0.98)	0.001*	0.99 (0.98, 1.01)	0.784
Seminal volume	0.87 (0.76, 1.01)	0.061	0.87 (0.74, 1.02)	0.088
Seminal concentration	1.00 (0.99, 1.01)	0.382	1.00 (0.99, 1.01)	0.517
Progressive sperm motility	0.99 (0.98, 1.00)	0.174	1.00 (0.98, 1.01)	0.760

The crude model had no adjustments for other covariates. The adjusted model was adjusted for all of the above variables. OR (95%CI) was used in the results and P < 0.05 was considered statistically significant. Bold fonts and * Statistically significant. a, adjusted; OR, odds ratio; CI, confidence interval; SDF, sperm DNA fragmentation; IVF, in vitro fertilization; ICSI, intracytoplasmic sperm injection; ET, embryo transfer; AMH, anti-mullerian hormone; AFC, antral follicle.

### Correlation between SDF level and CA rate in miscarried conceptus for different cycle types and maternal age groups

**Figure 3:**
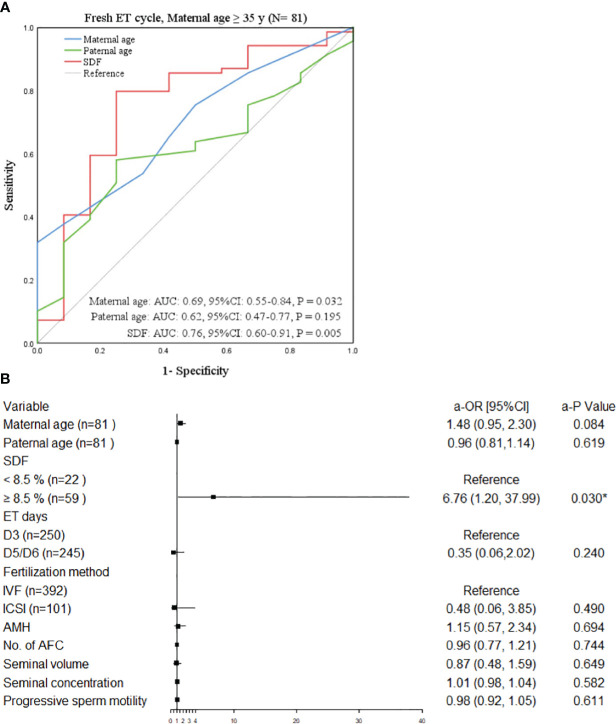
Factors related to chromosomal abnormalities in maternal age ≥35 years undergoing fresh ET. **(A)** The ROC curve of the subgroup of advanced women aged ≥35 years undergone fresh ET cycle, *P* < 0.05 was considered statistically significant. **(B)** The forest plot of the multivariate Logistic regression analysis results of the subgroup of elderly women aged ≥35 years treated with ET. All variables above were adjusted. OR (95%CI) was used in the results and *P* < 0.05 was considered statistically significant. * Statistically significant. OR, odds ratio; CI, confidence interval; SDF, sperm DNA fragmentation; IVF, *in vitro* fertilization; ICSI, intracytoplasmic sperm injection; ET, embryo transfer; AMH, anti-mullerian hormone; No., number; AFC, antral follicle.

The study population was divided into four groups according to ET type and maternal age. After adjusting the confounding variables, elevated SDF level was significantly correlated with the increase of aneuploidy rate in miscarried conceptus only in the advanced maternal age group who underwent fresh ET treatment (a-OR: 1.14 [1.00–1.29], a-*P* = 0.045, **Table 3**). The ROC curve showed that 8.5% was the most appropriate threshold for SDF related to aneuploidy rate in this group (AUC = 0.76 [0.60–0.91], *P* = 0.005, **Figure 3A**). After grouping SDF according to 8.5% and adjusting the previously mentioned variables, couples with paternal SDF ≥ 8.5% were 6.76 times more likely to have embryo aneuploid-related miscarriage than those with SDF < 8.5% (a-OR: 6.76 [1.20–37.99], a-*P* = 0.030, **Figure 3B**).

**Table 3:** Multivariable Logistic analyses of factors related to chromosomal aneuploidy in miscarried conceptus in different groups.

Variable	Fresh ET cycle				Frozen ET cycle			
	Maternal age < 35 y (n = 158)		Maternal age ≥ 35 y (n = 81)		Maternal age < 35 y (n = 196)		Maternal age ≥ 35 y (n = 60)	
	a-OR (95% CI)	a-*P*	a-OR (95% CI)	a-*P*	a-OR (95% CI)	a-*P*	a-OR (95% CI)	a-*P*
Maternal age	0.93 (0.79, 1.10)	0.413	1.49 (0.92, 2.42)	0.106	1.02 (0.91, 1.15)	0.693	1.24 (0.89, 1.72)	0.198
Paternal age								
20-29 y (n=151)	0.77 (0.32, 1.84)	0.550	6.56 (0.40, 108.28)	0.189	1.67 (0.82, 3.39)	0.160	NA	NA
30-39 y (n=284)	Reference		Reference		Reference		Reference	
≥ 40 y (n=60)	3606230278	1.000	2.30 (0.19, 28.50)	0.516	2.86(0.44, 18.54)	0.269	1.02 (0.23, 4.32)	0.983
SDF	1.04 (0.99, 1.08)	0.061	1.14 (1.00, 1.29)	0.045*	1.01 (0.98, 1.04)	0.545	1.03 (0.96, 1.09)	0.421
ET days								
D3 (n=250)	Reference		Reference		Reference		Reference	
D5/D6 (n=245)	0.48 (0.21, 1.08)	0.077	0.20 (0.03, 2.62)	0.118	0.69 (0.36, 1.34)	0.269	1.04 (0.29, 3.75)	0.950
Fertilization method								
IVF (n=392)	Reference		Reference		Reference		Reference	
ICSI (n=101)	0.78 (0.27, 2.25)	0.649	0.32 (0.04, 2.62)	0.288	0.71 (0.34, 1.51)	0.376	1.04 (0.16, 6.61)	0.970
AMH	1.57 (1.21, 2.04)	0.001*	1.37 (0.68, 2.76)	0.375	0.95 (0.85, 1.06)	0.324	1.13 (0.74, 1.74)	0.568
Number of AFC	0.90 (0.83, 0.98)	0.018*	0.94 (0.72, 1.22)	0.629	1.02 (0.95, 1.10)	0.554	0.98 (0.85, 1.14)	0.827
Seminal volume	0.97 (0.73, 1.26)	0.802	0.81 (0.443, 1.52)	0.509	0.83 (0.65, 1.07)	0.157	0.84 (0.50, 1.40)	0.497
Seminal concentration	1.01 (0.99, 1.02)	0.808	1.00 (0.98, 1.03)	0.808	1.00 (0.99, 1.01)	0.877	1.01 (0.99, 1.03)	0.436
Progressive sperm motility	0.99 (0.96, 1.02)	0.679	1.02 (0.93, 1.10)	0.733	0.99 (0.97, 1.02)	0.799	1.04 (0.97, 1.11)	0.285

All variables above were adjusted. OR (95%CI) was used in the results and P < 0.05 was considered statistically significant. Bold fonts and * Statistically significant. a, adjusted; OR, odds ratio; CI, confidence interval; SDF, sperm DNA fragmentation; IVF, in vitro fertilization; ICSI, intracytoplasmic sperm injection; ET, embryo transfer; AMH, anti-mullerian hormone; AFC, antral follicle.

### Correlation between elevated SDF and CA rate in miscarried conceptus

**Figure 4:**
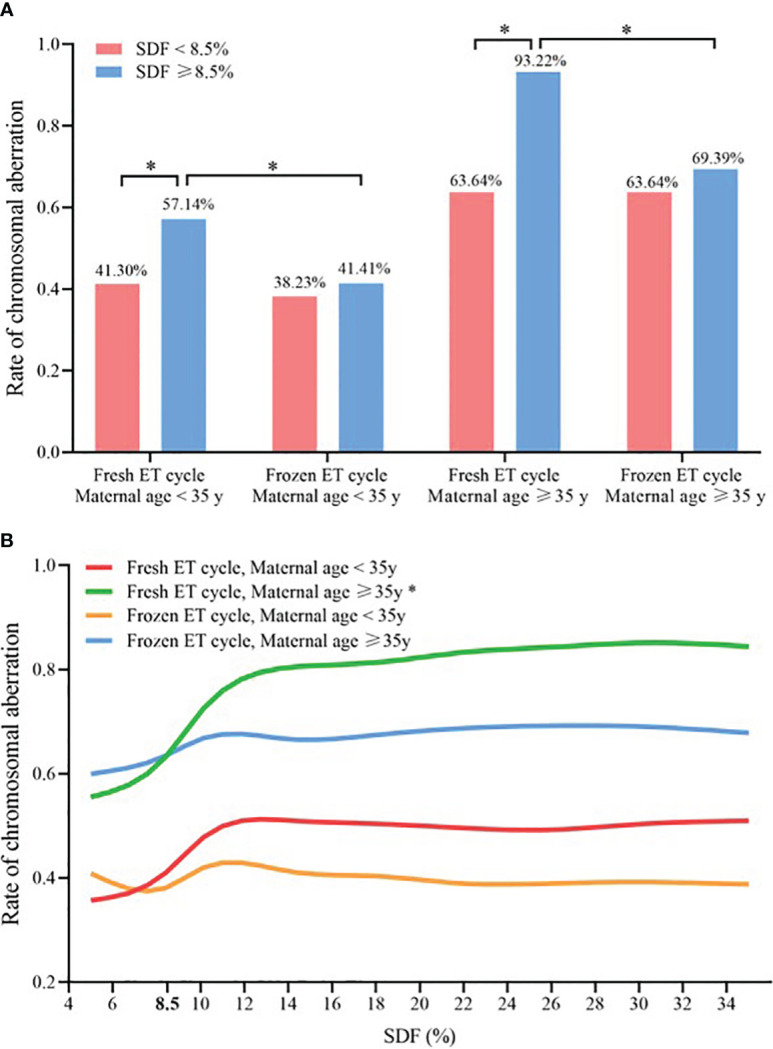
Comparison of chromosomal abnormalities in different ET strategies and maternal age groups **(A)** Chromosomal aneuploidy rates at SDF < 8.5% and ≥8.5% in the four subgroups, * Statistically significant. **(B)** Association between SDF and chromosomal aneuploidy rate of embryos in four subgroups, and maternal age, paternal age, ET days, fertilization method, AMH, number of antral follicle, seminal volume, seminal concentration and progressive sperm motility were adjusted. * Statistically significant, *P* < 0.05. SDF, sperm DNA fragmentation; ET, embryo transfer.

In women aged ≥ 35 years undergoing fresh ET cycle, the aneuploidy rate of chromosomes with SDF ≥ 8.5% was significantly higher than that with SDF < 8.5% (93.32% vs. 63.64%, P < 0.050, **Figure 4A**). And only for women aged ≥ 35 years undergoing fresh ET cycle, the CA rate of miscarried conceptus increased significantly with the increase of SDF in the adjusted model, achieving the maximum growth rate at SDF = 8.5% (green line: a-OR: 1.13 [1.01–1.27], a-*P* = 0.043), while this trend was not showed in the other three groups (red line: a-OR: 1.04 [0.99–1.08], a-*P* = 0.068; orange line: a-OR: 1.01 [0.98–1.04], a-*P* = 0.691; blue line: a-OR: 1.02 [0.97–1.09], a-*P* = 0.387, **Figure 4B**).

## Discussion

Previous studies on paternal SDF and embryo quality and chromosomal status are few and inconsistent. In this retrospective cohort study, we found a CVS aneuploidy rate of 54.95%, with trisomy being the most common, followed by abnormal chromosome structure. We also investigated the association between SDF and the CA rate of miscarried chorionic villi in patients after IVF/ICSI and demonstrated that elevated SDF, maternal age, paternal age and fresh/frozen ET cycle significantly correlated with the CA rate in miscarried conceptus, and for women of advanced age (≥ 35 years) who underwent fresh ET cycle, the use of sperm with paternal SDF ≥ 8.5% dramatically increased the risk of aneuploidy in miscarried products.

The mechanisms by which sperm affect the chromosomal integrity of embryos and the occurrence of miscarriage have not been clarified. Normal fertilization might not be impeded by high levels of sperm DNA fragmentation (29), however, it might jeopardize zygote development, embryo implantation and embryo healthy through genetic and epigenetic components of damaged chromatin (30, 31). Gawecka et al. (32) found that sperm DNA damage might lead to slowed paternal DNA replication or degradation after the S phase, ultimately delaying or stalling embryonic development. Two animal studies also indicated that sperm with damaged DNA might induce the fragmentation and random distribution of paternal chromosome in two-cell–stage embryos and lead to direct unequal cleavage divisions, segregation errors and the formation of haploid and uniparental cells, and specifical sperm fragment contributed to the chaotic aneuploidy of embryos (33, 34). This suggested that the late paternal effect caused by sperm DNA damage may be a key factor affecting embryonic genome integrity, leading to early abortion after activation of the male parent gene at the eight-cell stage (35). Our results showed that elevated SDF still contributed to increased aneuploidy rate in miscarried conceptus after adjusting for confounding factors that might affect embryonic euploidy. Although the potential mechanism is unclear, our results are supported by above-mentioned studies. This study provided a new perspective for the diagnosis and treatment of the association between high male SDF and miscarriage, although further studies are needed to explore the mechanism.

Advanced female age and fresh ET cycle have been proven to be closely related to high embryo chromosomal aneuploidy rate and low livebirth rate (36, 37). Li et al. (26) had found CA rate of miscarried conceptus in women ≥ 35 years old and fresh ET increased by 1.75 times and 0.78 times, respectively. These results are consistent with those of our study. We speculate that this probably owing to the occurrence of aneuploidy caused by epigenetic abnormalities (38), cell cycle checkpoint and meiotic recombination abnormalities (39), mitochondrial energy metabolism disorders (40), and telomere shortening (41) in oocytes during female aging. Moreover, low implantation rate and high miscarriage rate caused by fresh ET had been considered to be related to intimal molecular biology and histological abnormalities caused by overexposure to drugs such as ovulation induction (42). FET can prevent this damage, effectively screen and reject the implantation of low-quality abnormal embryos (43), and reduce the abortion rate of aneuploid embryos.

Interestingly, although we found a positive correlation between SDF and aneuploidy of miscarried CVS in the whole population, when we divided the population into four subgroups according to the maternal age (< 35 or ≥ 35 years old) and ET strategies (fresh ET or FET), only women of advanced age who underwent fresh ET cycle showed a facilitative effect of elevated SDF on aneuploid-related miscarriage. At the same time, the ROC curve showed that 8.5% was an appropriate threshold to predict the risk of aneuploid-related miscarriage in this subgroup, and when paternal SDF ≥ 8.5%, the risk of aneuploid-related miscarriage was 6.76 times higher than that of paternal SDF of < 8.5%. This result is consistent with some previous studies analyzing SDF and laboratory and clinical outcomes, but the threshold is obviously lower. Setti et al. (13) performed a retrospective study of 540 couples treated with ICSI and found that injection of sperm from samples with SDF ≥ 30% in women ≥ 40 years significantly decreased D3 good-quality embryo rate, blastocyst formation rate, implantation rate and clinical pregnancy rate, while increased miscarriage rate. Another study of young RIF patients under ICSI-PGS showed a higher CA rate in SDF > 20% group, especially in the trisomy rate of chromosomes 16 and 20 (5). Horta et al. (29) fertilized young and old mouse oocytes to mouse sperm with different degrees of SDF (≤ 10%, 11-30% and > 30% produced by different doses of radiation), and found the effect of high sperm DNA damage on embryonic development was related to the decrease of DNA repair ability of aged oocytes. There has been no international consensus on the threshold of the effect of SDF on male fertility, although most studies suggest that SDF ≥ 20% or SDF ≥ 30% were more likely to lead to male infertility and adverse pregnancy outcomes (44), our results showed that for women of advanced age (≥ 35 years), the threshold of SDF promoting aneuploid-related miscarriage might be lower than previously thought, which supported the hypothesis that the DNA repair ability of oocytes decreases with female senescence (13, 45).

Numerous studies have confirmed that oocytes had active DNA repair capability, in contrast to sperm. All of DNA repair pathways, including base excision repair, nucleotide excision repair, post replication repair, mismatch repair, double strand break repair and DNA damage response have already been proved to be maternal sources during fertilization and before embryonic genome activation (29, 46, 47). We did not observe a contribution of SDF to aneuploid-related miscarriage in young maternal groups, which suggested that oocytes could effectively repair patrilineal DNA damage. However, female oocytes are produced during the fetal period (48), and female senescence re oocyte senescence, and at this time, the transcripts and proteins stored in oocytes are reduced (49, 50). Compared with young oocytes, the DRA of aging oocytes is greatly decreased, resulting in loss of ability to cope with SDF, and then has a serious negative impact on embryonic development. Zygotes fertilized by DNA-damaged sperm have the potential to develop continuously under the DNA repairment of oocytes, whereas the lesion of DNA repair mechanism caused by oocyte senescence leads to sperm genetic material damage above a low threshold (e.g. 8.5%) that cannot be effectively repaired, resulting in early embryonic development defects, such as the formation of aneuploid embryos (51). Additionally, because of the application of fresh ET cycle, the endometrium function is relatively poor, with a low ability of screening and rejection of abnormal embryos (26, 43). Even though these aneuploid embryos escape the screening of endometrium and successfully implanted, they still failed to develop normally in the later stage, eventually leading to miscarriage.

We also found that males aged ≥40 years had an increased CA risk of CVS compared with those aged 30–39 years. This probably due to the accumulating cellular damage and exposure to environmental toxins in parallel with increasing age, leading to epigenetics changes, DNA methylation defects, increased reactive oxygen species damage, shorter telomere length, and damage of mitotic and meiotic mismatch repair mechanisms (52, 53), ultimately damaging the integrity of embryonic chromosomes and increasing the risk of the formation rate of aneuploidy and mosaic embryos (54, 55). Nevertheless, this phenomenon disappeared after grouping the population. We speculate that this may be due to a statistical deviation caused by a decrease in the proportion of men aged ≥40 years in each subgroup. Besides, the contribution of advanced paternal age may not be as obvious as that of advanced maternal age, which may mask the impact of paternal age. Future work should expand the sample size to determine the contribution of greater paternal age to this risk.

To our knowledge, this is the first study to show the influence of paternal SDF on the rate of chromosomal aberrations in miscarried CVS. We used strict inclusion and exclusion criteria to reduce confounding risk factors (56), while adjusting the variant model for correlation analysis to increase statistical rigor. However, our study also has several limitations. First, retrospective design data obtained from a single center with a small sample size. Since there remains no prospective multicenter study of the effects of SDF on embryonic aneuploidy, we look forward to future high-quality studies with relevant content to provide clinicians with more and more reliable evidence. Second, even though the data collection was reviewed by medical staff, errors in the data collection and follow-up process could not be avoided. Third, patients may be subject to inclusion bias due to missing data on environment, lifestyle, occupational characteristics, psychological status, and family factors. Nevertheless, the findings of this study are still valuable. For couples with advanced maternal age and high paternal SDF index, preimplantation genetic screening and FET may be worth considering to avoid transfer of aneuploid embryos. Further research is required to determine the risks of developing such a clinical strategy.

## Conclusion

In conclusion, elevated SDF are significantly associated with an elevated CA rate in miscarried conceptus. In a population of women ≥ 35 years of age undergoing fresh ET cycle, fertilization of older oocytes using sperm with high levels of SDF significantly increases the risk of miscarriage of aneuploid fetuses, especially the risk of single chromosomal trisomy. For women of advanced age treated with ART, sperm with high SDF levels should be used with vigilance during ART to avoid compromising pregnancy outcomes.

## Data availability statement

The original contributions presented in the study are included in the article/supplementary material. Further inquiries can be directed to the corresponding author.

## Author contributions

WF: Writing – review & editing, Data curation, Writing – original draft. QC: Writing – original draft, Writing – review & editing. ZB: Writing – review & editing. HS: Writing – review & editing. QY: Writing – review & editing. LH: Writing – review & editing, Funding acquisition.
